# Assessing the usefulness of a newly proposed metabolic score for visceral fat in predicting future diabetes: results from the NAGALA cohort study

**DOI:** 10.3389/fendo.2023.1172323

**Published:** 2023-07-19

**Authors:** Ruijuan Yang, Maobin Kuang, Jiajun Qiu, Changhui Yu, Guotai Sheng, Yang Zou

**Affiliations:** ^1^ Department of Endocrinology, Jiangxi Provincial People’s Hospital, Medical College of Nanchang University, Nanchang, Jiangxi, China; ^2^ Jiangxi Cardiovascular Research Institute, Jiangxi Provincial People’s Hospital, The First Affiliated Hospital of Nanchang Medical College, Nanchang, Jiangxi, China; ^3^ Department of Cardiology, Jiangxi Provincial People’s Hospital, Medical College of Nanchang University, Nanchang, Jiangxi, China; ^4^ Jiangxi Provincial Geriatric Hospital, Jiangxi Provincial People’s Hospital, The First Affiliated Hospital of Nanchang Medical College, Nanchang, Jiangxi, China

**Keywords:** diabetes, METS-VF, predictive power, time-dependent ROC analysis, visceral adiposity

## Abstract

**Objective:**

Visceral adipose tissue assessment holds significant importance in diabetes prevention. This study aimed to explore the association between the newly proposed Metabolic Score for Visceral Fat (METS-VF) and diabetes risk and to further assess the predictive power of the baseline METS-VF for the occurrence of diabetes in different future periods.

**Methods:**

This longitudinal cohort study included 15,464 subjects who underwent health screenings. The METS-VF, calculated using the formula developed by Bello-Chavolla et al., served as a surrogate marker for visceral fat obesity. The primary outcome of interest was the occurrence of diabetes during the follow-up period. Established multivariate Cox regression models and restricted cubic spline (RCS) regression models to assess the association between METS-VF and diabetes risk and its shape. Receiver operating characteristic (ROC) curves were used to compare the predictive power of METS-VF with body mass index (BMI), waist circumference (WC), waist-to-height ratio (WHtR), and visceral adiposity index (VAI) for diabetes, and time-dependent ROC analysis was conducted to assess the predictive capability of METS-VF for the occurrence of diabetes in various future periods.

**Results:**

During a maximum follow-up period of 13 years, with a mean of 6.13 years, we observed that the cumulative risk of developing diabetes increased with increasing METS-VF quintiles. Multivariable-adjusted Cox regression analysis showed that each unit increase in METS-VF would increase the risk of diabetes by 68% (HR 1.68, 95% CI 1.13, 2.50), and further RCS regression analysis revealed a possible non-linear association between METS-VF and diabetes risk (*P* for non-linearity=0.002). In addition, after comparison by ROC analysis, we found that METS-VF had significantly higher predictive power for diabetes than other general/visceral adiposity indicators, and in time-dependent ROC analysis, we further considered the time-dependence of diabetes status and METS-VF and found that METS-VF had the highest predictive value for predicting medium- and long-term (6-10 years) diabetes risk.

**Conclusion:**

METS-VF, a novel indicator for assessing visceral adiposity, showed a significantly positive correlation with diabetes risk. It proved to be a superior risk marker in predicting the future onset of diabetes compared to other general/visceral adiposity indicators, particularly in forecasting medium- and long-term diabetes risk.

## Introduction

Diabetes is one of the most common chronic diseases that endangers the physical health of the world population and cause disability and death (1, 2). The treatment and management of diabetic patients heavily burden the world’s healthcare systems and have become an important global public health challenge (3, 4). Under the background that diabetes currently cannot be completely cured, the early identification of people at risk of developing diabetes and primary prevention of diabetes are of great public health importance (5).

Obesity is an important risk factor for the development and progression of diabetes (6), and obese people are usually at a higher risk for diabetes. Notably, compared to general adiposity due to increased subcutaneous fat, visceral adiposity is more harmful to the organism, especially fat deposits in organs such as the liver and skeletal muscle, which cause more pronounced hepatic and peripheral insulin resistance thereby leading to the development of metabolic diseases such as diabetes (7–9). The gold standard measure for clinical assessment of visceral adiposity is magnetic resonance imaging (MRI), but it is not suitable for diabetes screening and clinical prevention in large populations due to its expensive testing costs and complex procedures (10). In addition, anthropometric abdominal adiposity indicators WC, WHtR, and waist-to-hip ratio can also indicate the risk of visceral adiposity, but they cannot accurately distinguish between abdominal visceral adipose tissue and subcutaneous adipose tissue (11).

METS-VF is a newly developed surrogate for assessing visceral adiposity that integrates demographic parameters (age and sex), anthropometric obesity parameters (BMI and WHtR), and glycemic lipid parameters [fasting plasma glucose (FPG), triglyceride (TG), and high-density lipoprotein cholesterol (HDL-C)]. It was developed by Bello-Chavolla OY et al. Following validation and comparison by Bello-Chavolla OY et al., METS-VF was found to provide a significantly superior estimate of human visceral adiposity compared to other commonly used surrogate indicators for abdominal adiposity. Moreover, it exhibited high agreement with gold standard measurements (12). Several subsequent observational studies have shown that METS-VF had good risk assessment/predictive power for metabolic diseases closely related to visceral adiposities such as chronic kidney disease, hypertension, and hyperuricemia (13–16). However, the correlation between METS-VF and diabetes risk has only been explored in a rural population in China (17), and the predictive power of baseline METS-VF for the future development of diabetes in the general population and the effect of temporal progression on the predictive power of METS-VF are currently unknown. Therefore, the current study comprehensively analyzed and compared the risk assessment/predictive ability of METS-VF for diabetes based on a larger sample size general population cohort and further explored the predictive power of METS-VF for the occurrence of diabetes in different future periods using time-dependent ROC analysis.

## Methods

### Study design and ethics approval

We conducted a retrospective cohort study of subjects in the NAGALA cohort (NAfld in the Gifu Area, Longitudinal Analysis) to assess the usefulness of the newly proposed METS-VF for predicting future diabetes. Information on the NAGALA cohort study was described in detail in a previously published article (18). In brief, the NAGALA cohort was established in 1994 and included a study sample of people who underwent health screenings at Murakami Memorial Hospital. Given that the vast majority of people who underwent health screenings at the hospital will have repeat screenings in the future, with 60% of these subjects receiving one or two health screenings per year, the NAGALA research project team conducted a long-term follow-up survey for future incident diabetes and incident non-alcoholic fatty liver disease. In a previously published article, Prof. Okamura reported that the NAGALA cohort study was approved by the Murakami Memorial Hospital ethics committee and written informed consent was obtained from all study participants, and that detailed data from the study were uploaded to the Dryad public database for sharing (19). The current study is a secondary analysis of the NAGALA study, and the subjects’ identifying information has been anonymized in the data set used. Therefore, the Ethics Committee of Jiangxi Provincial People’s Hospital waived the process of obtaining written informed consent for the current study, approved the protocol of the current study, and supervised the entire process of the current study. See STROBE statement (S1Text).

### Study population

**Figure 1:**
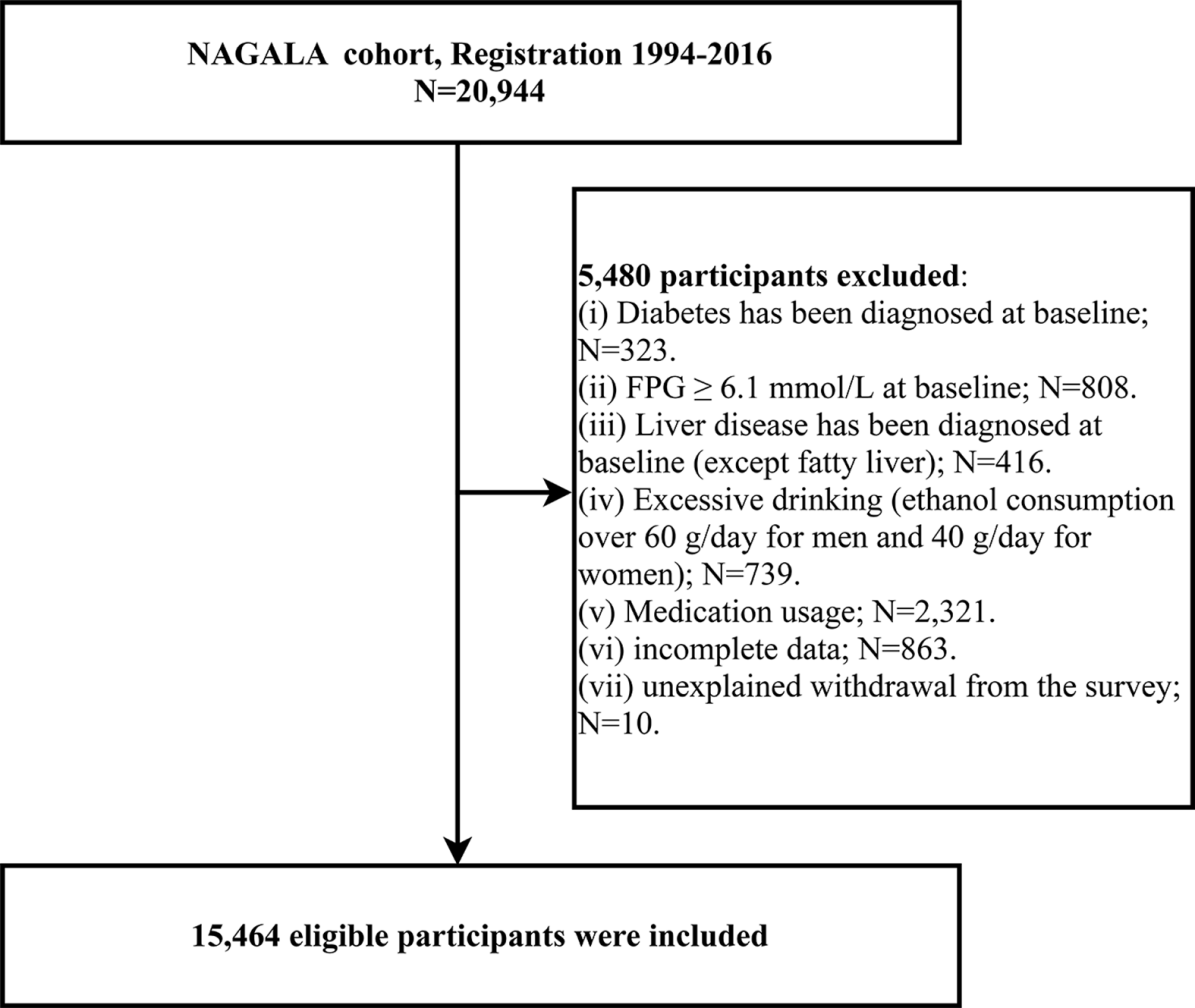
Flowchart of the selection process of study subjects.

The current study extracted data from the NAGALA cohort of 20,944 subjects who underwent health screenings between May 1994 and December 2016. We further excluded subjects with the following conditions according to the study objectives: (1) At baseline, 323 who had been diagnosed with diabetes, 416 with liver disease (other than fatty liver), and 808 with FPG ≥6.1mmol/L; (2) 2,321 who were taking medications at baseline, 739 with excessive alcohol consumption (20), and 863 with incomplete data; (3) 10 who withdrew from the study during follow-up for unknown reasons. Ultimately, 15,464 subjects were included in the current study for analysis, and the detailed flow chart was shown in **Figure 1**.

### Baseline data collection and definition of diabetes

Standardized trained medical examiners collected basic information on sex, age, smoking and drinking status, and exercise habits by means of a questionnaire. Smoking status was defined using never, past and current smoking; drinking status was defined as non/small, light, moderate, and heavy drinking based on the subject’s weekly alcohol consumption in the month prior to study participation (20); and having an exercise habit was defined as the subject having at least one physical activity per week. Anthropometric indicators of height, weight, WC, and systolic and diastolic blood pressure (SBP and DBP) were measured indoors with subjects wearing light clothing and no shoes using standard methods. Fatty liver diagnosis was based on the evaluation of liver contrast and brightness in abdominal ultrasound images by gastroenterologists (18, 21). In addition, forearm venous blood samples were drawn from subjects after fasting for at least 8 hours and sent to a standard laboratory, and then using an automated biochemical analyzer measured concentrations of biochemical parameters such as aspartate aminotransferase (AST), HDL-C, alanine aminotransferase (ALT), glycosylated hemoglobin (HbA1c), FPG, gamma-glutamyl transferase (GGT), TG, and total cholesterol (TC).

### Primary outcome

The incidence of diabetes among the subjects during the follow-up period was considered the primary outcome in the current study. According to the American Diabetes Association criteria, diabetes was defined as HbA1c ≥6.5% or FPG ≥7.0 mmol/L measured during follow-up or self-reported diabetes (verified through the examination of subjects’ medical records or blood glucose measurements) by the subject (22).

### Calculation formulas for METS-VF, BMI, WHtR, and VAI

METS-VF = 4.466 + 0.011*[(Ln((Ln((2 * FPG) + TG) * BMI)/(Ln(HDL- C))))^3^] +3.239*[(Ln(WHtR))^3^] + 0.319 * sex + 0.594 * (Ln(age)) (12). Note: sex in the METS-VF calculation formula was a binary response variable (men=1, women=0).


$$BMI=weight(kg){\left[height(m)\right]}^{2}$$

WHtR=WC(cm)/height(cm)


VAI(men)=(WC/(39.68+(1.88*BMI))*(TG/1.03)*(1.31/HDL−C)

 (23)


VAI(women)=(WC/(36.58+(1.89*BMI))*(TG/0.81)*(1.52/HDL−C)

 (23)

### Statistical analysis

All statistical analyses for the current study were done on R Language 3.4.3 and Empower(R) 2.0 software and were set to be significant at two-sided *P<*0.05. METS-VF values were calculated and all subjects were grouped according to quintiles of METS-VF values [Quintile 1 (Q1)<5.03, Q2 (5.03 to 5.58), Q3 (5.58 to 6.00), Q4 (6.00 to 6.42), Q5 ≥6.42] using the quantile function. Described the baseline data of the subjects according to the quintiles of METS-VF, and chose different description methods and comparison methods between groups according to the type of data; among them, continuous variables with normal and skewed distribution were described as mean (standard deviation) and median (interquartile range), respectively, and comparisons between groups were performed using one-way ANOVA and Kruskal-Willis H test, respectively, while categorical variables were described as frequencies (%), and comparisons between groups were made using chi-square tests. In addition, we used Kaplan-Meier curves to describe the cumulative hazard of developing diabetes in each METS-VT quintile during the follow-up period and subsequently examined the differences between the groups using log-rank tests and finally made a preliminary determination of whether the proportional hazards assumption for establishing multivariate Cox regression models was met based on the results of Kaplan-Meier analysis (24).

To clarify the association between baseline indicators and diabetes risk and to initially explore the association of METS-VF with diabetes risk, we first estimated the hazard ratio (HR) and 95% confidence interval (CI) for each baseline indicator associated with diabetes risk using univariate Cox regression analysis. Subsequently, we checked for collinearity between all covariates and METS-VF by multiple linear regression analysis and excluded collinear variables with a final variance inflation factor greater than 5 from later model adjustments (25). According to the recommendations of the STROBE guidelines (26), we established four stepwise adjusted multivariate Cox regression models; Model 1 was adjusted for age, sex, and BMI; Model 2 considered the potential effects of fatty liver and lifestyle-related factors (smoking and drinking status and exercise habits) on the basis of Model 1; Model 3 was further adjusted for liver function-related indicators (ALT, AST, and GGT); finally, Model 4 continued to adjust for SBP, TG, HDL-C, TC, and HbA1c on the basis of Model 3. We incorporated METS-VF into 4 multivariate Cox regression models as continuous variables and categorical variables in quintiles, respectively, and calculated trends associated with diabetes risk based on the median of METS-VF quintiles in the models. Furthermore, to detect any possible linear or non-linear dependence between METS-VF, BMI, WC, WHtR, and VAI and diabetes risk, we utilized a 4-knot RCS model to fit dose-response curves for these variables at the 5th, 35th, 65th, and 95th percentiles. Prior to plotting the dose-response curves, we also conducted separate collinearity screenings to examine the presence of collinearity between BMI, WC, WHtR, VAI, and other covariates. Based on the results of the collinearity screening analysis, we adjusted for covariates that showed no collinearity with the respective obesity indicators in the RCS regression models.

ROC curves were constructed and the area under the curves (AUCs) was calculated to assess the predictive power of baseline METS-VF and several traditional visceral adiposity indicators, WC, WHtR, VAI, and BMI, for diabetes, and the differences in predictive power between METS-VF and the other indicators were compared using the DeLong test (27). Additionally, to assess the effect of time factors on the ability of METS-VF to predict the future occurrence of diabetes, we also calculated the AUCs, optimal thresholds, sensitivity, and specificity of baseline METS-VF for predicting the occurrence of diabetes at each time point from 2 to 12 years in the future using time-dependent ROC analysis. Subsequently, we evaluated the calibration of the predictive model by plotting calibration curves to assess the agreement between predicted probabilities and observed probabilities; internal validation was conducted using the bootstrap algorithm with 1,000 repetitions (28).

## Results

### Baseline characterization

After screening the study population according to inclusion and exclusion criteria, a total of 15,464 subjects were eventually enrolled in the current study (**Figure 1**), with a mean age of 43.71 years, of which 54.51% were men. **Table 1** groups all subjects according to the quintiles of METS-VF and describes and compares the baseline information of each group; we found that with the increase of METS-VF quintile, the proportion of subjects who were men, fatty liver patients, alcohol drinkers, and current and past smokers all gradually increased, while the proportion of those with an exercise habit gradually decreased (All *P<*0.001). Regarding the anthropometric indicators and biochemical parameters of the subjects, except for HDL-C levels, which decreased with the increase of METS-VF quintile, the levels of other indicators such as age, height, weight, BMI, WC, ALT, AST, GGT, TC, TG, FPG, HbA1c, SBP, and DBP increased with the increase of METS-VF quintile (All *P<*0.001).

**Table 1:** Baseline characteristics of subjects and incidence of diabetes grouped according to METS-VF quintiles.

	METS-VF quintiles					*P*-value
	Quintile 1 (< 5.03)	Quintile 2 (5.03 to 5.58)	Quintile 3 (5.58 to 6.00)	Quintile 4 (6.00 to 6.42)	Quintile 5 (≥ 6.42)	
Subjects, n	3091	3090	3091	3090	3091	
Sex						<0.001
Women	2405 (77.81%)	1834 (59.35%)	1297 (41.96%)	910 (29.45%)	588 (19.02%)	
Man	686 (22.19%)	1256 (40.65%)	1794 (58.04%)	2180 (70.55%)	2503 (80.98%)	
Age, year	38.00 (35.00-43.00)	40.00 (36.00-47.00)	42.00 (37.00-49.00)	45.00 (39.00-52.00)	49.00 (41.00-55.00)	<0.001
Height, m	1.63 (0.08)	1.64 (0.09)	1.66 (0.09)	1.67 (0.08)	1.67 (0.08)	<0.001
Weight, kg	49.66 (6.32)	55.36 (7.41)	60.22 (8.10)	65.10 (8.83)	72.82 (10.95)	<0.001
BMI, kg/m^2^	18.73 (1.45)	20.51 (1.42)	21.82 (1.52)	23.39 (1.73)	26.13 (2.76)	<0.001
WC, cm	65.09 (3.91)	71.33 (3.40)	76.32 (3.41)	80.97 (3.77)	88.63 (6.16)	<0.001
ALT, U/L	13.00 (11.00-17.00)	14.00 (11.00-18.00)	16.00 (12.00-22.00)	19.00 (15.00-26.00)	24.00 (18.00-34.00)	<0.001
AST, U/L	16.00 (13.00-19.00)	16.00 (13.00-19.00)	17.00 (14.00-21.00)	18.00 (15.00-22.00)	20.00 (16.00-25.00)	<0.001
GGT, U/L	12.00 (9.00-15.00)	13.00 (10.00-17.00)	15.00 (11.00-21.00)	18.00 (13.00-27.00)	23.00 (16.00-34.00)	<0.001
HDL-C, mmol/L	1.71 (0.38	1.59 (0.39)	1.47 (0.37)	1.34 (0.35)	1.20 (0.30)	<0.001
TC, mmol/L	4.82 (0.81)	4.95 (0.81)	5.13 (0.84)	5.27 (0.86)	5.46 (0.85)	<0.001
TG, mmol/L	0.50 (0.37-0.69)	0.59 (0.42-0.81)	0.75 (0.53-1.05)	0.93 (0.64-1.33)	1.19 (0.84-1.72)	<0.001
FPG, mg/dL	88.77 (6.92)	90.95 (6.89)	93.06 (6.90)	95.02 (6.88)	97.01 (6.63)	<0.001
HbA1c, %	5.10 (0.30)	5.13 (0.30)	5.16 (0.31)	5.20 (0.32)	5.27 (0.34)	<0.001
SBP, mmHg	105.32 (12.22)	109.63 (12.55)	114.21 (13.06)	118.55 (13.50)	124.75 (15.23)	<0.001
DBP, mmHg	65.14 (8.37)	67.99 (8.88)	71.17 (9.47)	74.62 (9.52)	78.97 (10.22)	<0.001
Exercise habits	510 (16.50%)	574 (18.58%)	594 (19.22%)	550 (17.80%)	478 (15.46%)	<0.001
Fatty liver	9 (0.29%)	77 (2.49%)	291 (9.41%)	761 (24.63%)	1599 (51.73%)	<0.001
Drinking status						<0.001
Non/small	2720 (88.00%)	2494 (80.71%)	2305 (74.57%)	2193 (70.97%)	2090 (67.62%)	
Light	213 (6.89%)	330 (10.68%)	397 (12.84%)	405 (13.11%)	409 (13.23%)	
Moderate	131 (4.24%)	214 (6.93%)	279 (9.03%)	324 (10.49%)	409 (13.23%)	
Heavy	27 (0.87%)	52 (1.68%)	110 (3.56%)	168 (5.44%)	183 (5.92%)	
Smoking status						<0.001
Never	2395 (77.48%)	2045 (66.18%)	1769 (57.23%)	1554 (50.29%)	1264 (40.89%)	
Past	273 (8.83%)	455 (14.72%)	604 (19.54%)	722 (23.37%)	895 (28.96%)	
Current	423 (13.68%)	590 (19.09%)	718 (23.23%)	814 (26.34%)	932 (30.15%)	
Diabetes incidence	11 (0.4%)	27 (0.9%)	41 (1.3%)	77 (2.5%)	217 (7.0%)	<0.001

Values were expressed as mean (SD) or medians (quartile interval) or n (%). BMI, body mass index; WC, Waist circumference; ALT, alanine aminotransferase; AST, aspartate aminotransferase; GGT, gamma-glutamyl transferase; HDL-C, high-density lipoprotein cholesterol; TC, total cholesterol; TG, triglyceride; HbA1c, hemoglobin A1c; FPG, fasting plasma glucose; SBP, systolic blood pressure; DBP, Diastolic blood pressure; METS-VF, Metabolic Score for Visceral Fat.

**Figure 2:**
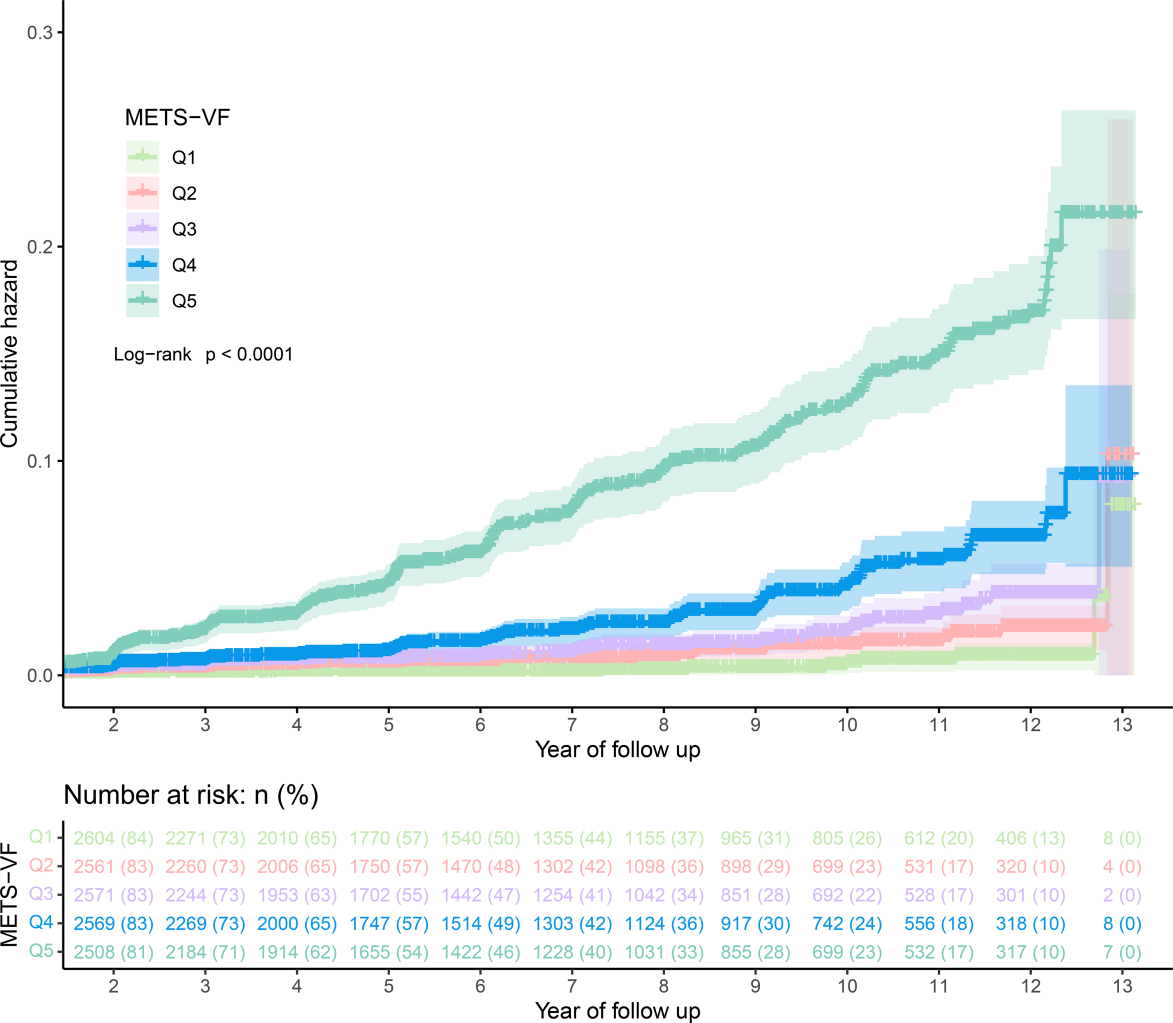
Kaplan-meier curve of METS-VF quartiles over time. METS-VF, Metabolic Score for Visceral Fat.

During a follow-up period of up to 13 years with an average duration of 6.13 years, a total of 373 individuals developed diabetes, resulting in an incidence rate of 39.88/10,000 person-years. Notably, the incidence rate of diabetes demonstrated a gradual upward trend across the quintiles of METS-VF. Specifically, the incidence rates for Q1-Q5 were 0.4%, 0.9%, 1.3%, 2.5%, and 7.0%, respectively. Moreover, we used Kaplan-Meier curves to describe the cumulative hazard of developing diabetes in each METS-VF quintile during the follow-up period (**Figure 2**), and the results also showed a progressive increase in the risk of developing diabetes with increasing METS-VF quintiles and no significant intersection of the curves (log-rank *P<*0.0001), which also suggested that our data followed the proportional hazard assumption.

### Association of METS-VF with diabetes

**Supplementary Table 1** shows the results of the univariate Cox regression analysis between baseline variables and diabetes risk, where we found that all baseline variables were significantly associated with diabetes risk (*P<*0.0001) except for exercise habits, which was borderline positive [(HR 0.76, 95% CI 0.56, 1.02), *P*=0.0641], where each unit increase in METS-VF increased the risk of diabetes by 414% (HR 5.14, 95% CI 4.27, 6.19). To further explore the independent association of METS-VF with diabetes risk, we included METS-VF as continuous and categorical variables, respectively, in four multivariate Cox regression models (**Table 2**), in which the non-collinear variables were adjusted stepwise while the collinear variables weight, WC, and DBP were excluded (**Supplementary Table 2**). When we preliminarily adjusted age, sex, and BMI in Model 1, we found that METS-VF as a continuous variable remained significantly positively correlated with diabetes risk (HR 2.81, 95% CI 1.92, 4.12), while as a categorical variable, taking Q1 as a reference, the risk of diabetes increased gradually with the increase of METS-VF quintile and the two were linearly correlated (*P*-trend<0.001). After further adjusting the fatty liver and lifestyle indicators (Model 2), and liver function-related parameters (Model 3), the HR of METS-VF associated with diabetes risk decreased slightly, while the direction and linear trend of the association remained unchanged. Ultimately, we additionally adjusted for SBP, TG, HDL-C, TC, and HbA1c in Model 4 and found that each unit increase in METS-VF would increase the risk of diabetes by 68% (HR 1.68, 95% CI 1.13, 2.50); in addition, Q5 still had the highest diabetes risk (HR 2.15, 95% CI 0.98, 4.70) with Q1 as a reference in Model 4, but the linear association between METS-VF quintiles and diabetes risk was not significant after trend test (*P*-trend=0.0946), which suggested that there may be a non-linear relationship between the two.

**Table 2:** Multivariable Cox regression analyses for the association between METS-VF and the incidence of diabetes.

	HR (95%CI)			
	Model 1	Model 2	Model 3	Model 4
METS-VF (continuous)	2.81 (1.92, 4.12)	2.75 (1.87, 4.03)	2.45 (1.67, 3.58)	1.68 (1.13, 2.50)
Quintile 1	Ref	Ref	Ref	Ref
Quintile 2	1.70 (0.83, 3.45)	1.70 (0.84, 3.46)	1.77 (0.87, 3.60)	1.63 (0.80, 3.33)
Quintile 3	2.01 (1.00, 4.02)	2.03 (1.01, 4.05)	2.05 (1.03, 4.10)	1.60 (0.79, 3.24)
Quintile 4	2.51 (1.26, 5.02)	2.52 (1.26, 5.03)	2.48 (1.24, 4.95)	1.67 (0.82, 3.40)
Quintile 5	4.08 (1.94, 8.60)	4.13 (1.96, 8.71)	3.81 (1.80, 8.06)	2.15 (0.98, 4.70)
*P*-trend	<0.001	<0.001	0.0001	0.0946

HR, Hazard ratio; CI, confidence interval; other abbreviations as in **Table 1**.

Model 1 adjusted for age, sex, and BMI.

Model 2 adjusted for age, sex, BMI, fatty liver, habits of exercise, smoking status, and drinking status.

Model 3 adjusted for age, sex, BMI, fatty liver, habits of exercise, smoking status, drinking status, ALT, AST, and GGT.

Model 4 adjusted for age, sex, BMI, fatty liver, habits of exercise, smoking status, drinking status, ALT, AST GGT, SBP, TG, HDL-C, TC, and HbA1c.

### Non-linear association between METS-VF, BMI, WC, WHtR and VAI and diabetes risk

**Figure 3:**
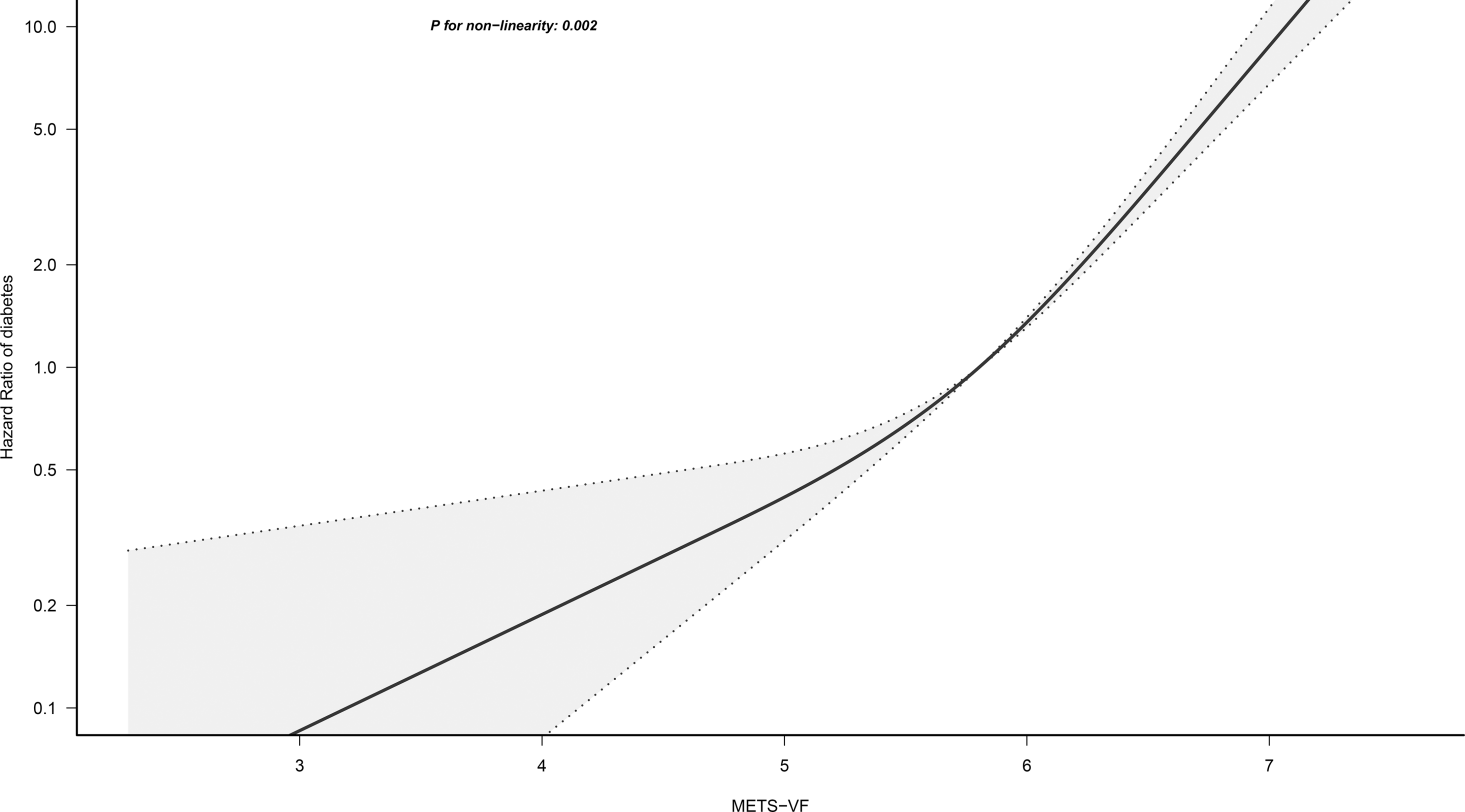
Restricted cubic spline analysis of METS-VF for the estimation of the risk of diabetes. METS-VF, Metabolic Score for Visceral Fat. Restricted cubic spline model adjusted for sex, age, fatty liver, height, BMI, exercise habits, ALT, AST, GGT, HDL-C, TC, TG, HbA1c, drinking status, smoking status, FPG, and SBP.

We employed a 4-knot RCS regression model to fit the dose-response curves for METS-VF, BMI, WC, WHtR, and VAI in relation to the risk of diabetes. Adjustments for non-collinear variables were made in the corresponding RCS regression models based on the results of collinearity analysis (**Supplementary Tables 2-6**). The RCS analysis revealed that the association between METS-VF and diabetes risk was non-linear (*P* for non-linearity=0.002) (**Figure 3**); when the METS-VF value was in the Q3 (5.58-6.00) interval, the slope of the curve increased significantly with the increase of METS-VF, implying that METS-VF had a stronger correlation with diabetes risk in the Q4 and Q5 intervals compared to the Q1 and Q2 intervals. Moreover, BMI, WC, WHtR, and VAI demonstrated similar shapes of association with the risk of diabetes, with evident threshold points on the curves, and all exhibiting non-linear correlations (**Supplementary Figures 1-4**; All *P* for non-linearity<0.05).

### Comparison of METS-VF with BMI, WC, WHtR, and VAI in predicting diabetes and time-dependent ROC analysis

**Table 3** shows the AUC, Sensitivity, Specificity, Positive predictive value and Negative predictive value (NPV) of METS-VF, BMI, WC, WHtR, and VAI for predicting diabetes. Overall, BMI, VAI, WC, WHtR, and METS-VF all had a good predictive performance for diabetes with AUC values of 0.73 (0.71, 0.76), 0.74 (0.71, 0.77), 0.74 (0.72, 0.77), 0.74 (0.72, 0.77), 0.77 (0.75, 0.80), respectively. After comparison, it was found that METS-VF had a significantly higher AUC value (0.77) than other indicators, showing the highest predictive accuracy for future diabetes risk (All *P<*0.05, DeLong test). In addition, all the aforementioned indicators of visceral obesity exhibited high NPV, with METS-VF having the highest NPV of 99.10%.

**Table 3:** Area under the ROC curve, Sensitivity, Specificity, PPV, and NPV of METS-VF, BMI, WC, VAI, and WHtR to predict diabetes.

	AUC	95%CI low	95%CI up	Specificity	Sensitivity	PPV	NPV
BMI*	0.7327	0.7068	0.7585	71.82%	62.73%	5.22%	98.73%
VAI*	0.7410	0.7145	0.7674	68.18%	71.58%	5.27%	98.98%
WC*	0.7424	0.7164	0.7685	71.63%	65.42%	5.39%	98.82%
WHtR*	0.7424	0.7167	0.7682	77.01%	60.32%	6.09%	98.74%
METS-VF	0.7731	0.7493	0.7969	65.07%	76.14%	5.12%	99.10%

ROC, receiver-operating characteristic curve; AUC, area under the ROC curve; PPV, positive predictive value; NPV, negative predictive value; VAI, visceral adiposity index; WHtR, waist-to-height index; CI, confidence interval; Other abbreviations as in **Table 1**; *, P<0.05 compared with METS-VF.

**Figure 4:**
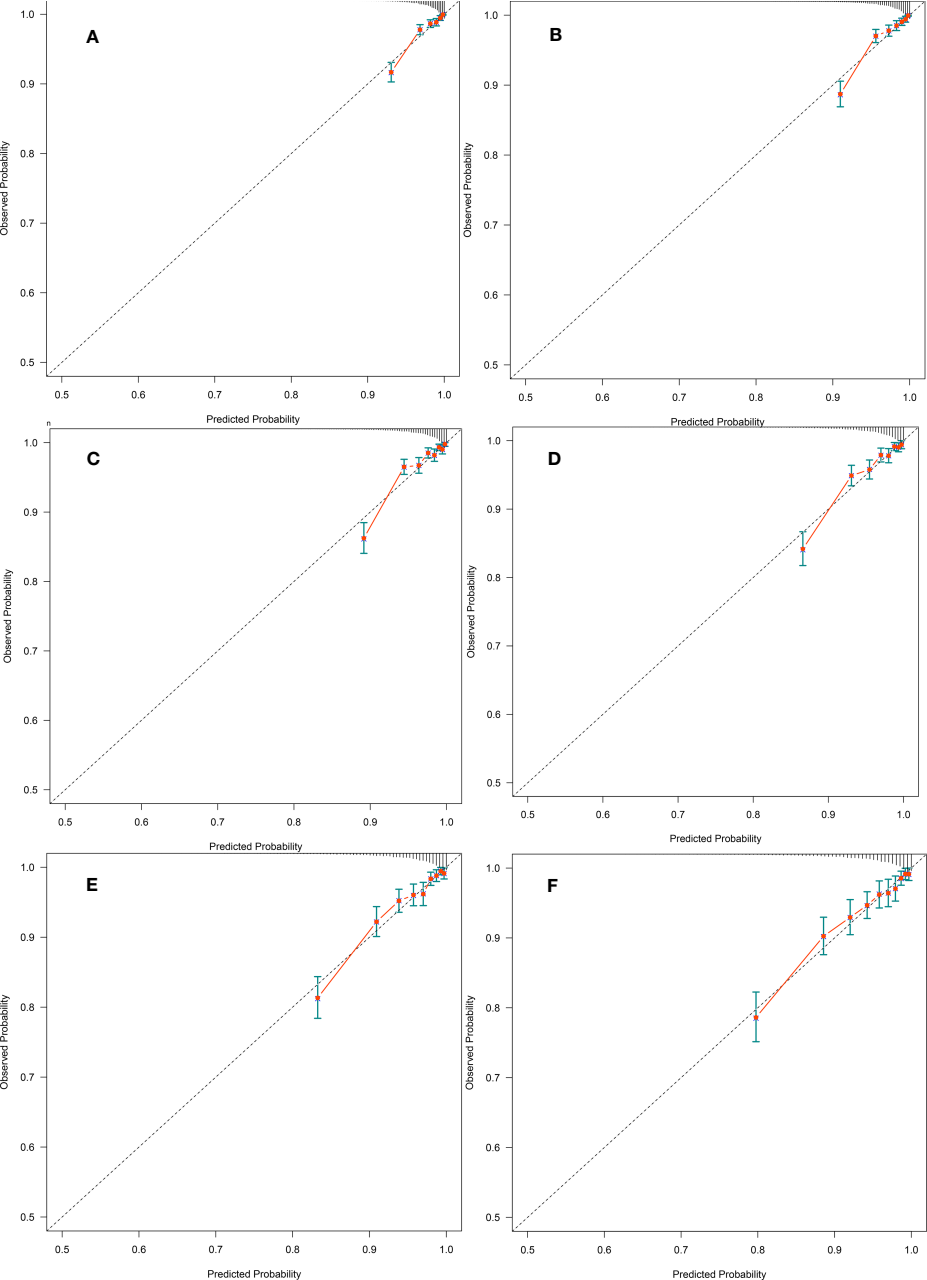
**(A-F)** were calibration curves of the prediction for diabetes event at year-7 to year-12, respectively. Dashed lines on the diagonal are reference lines.

This study also used time-dependent ROC analysis to further explore the predictive power of METS-VF for each time point over the next 2-12 years regarding the occurrence of diabetes (**Table 4**). Additionally, the calibration ability of METS-VF in predicting long-term diabetes risk (7-12 years) was evaluated using calibration curves (**Figure 4**). The results of the analysis showed that the predictive power of METS-VF for future diabetes risk gradually increased from the 2nd year of follow-up, until the AUC reached the highest value of 0.79 at the 7th and 8th year of follow-up, and then gradually decreased from the 9th year; specifically, METS-VF had higher AUC values (>0.77) and more stable prediction thresholds (6.03-6.37) for predicting diabetes over the next 6-10 years, which was an ideal risk marker for predicting the occurrence of diabetes in the future medium- and long-term. Furthermore, the calibration curves in **Figure 4** demonstrated that the predicted diabetes risk by METS-VF aligned well with the observed diabetes risk in the year-7 to year-12 period. This indicated that METS-VF had a reliable predictive accuracy for diabetes.

**Table 4:** Areas under the time-dependent ROC curves, Best thresholds, Sensitivity, and Specificity for METS-VF predicting future diabetes risk.

	2-years	3-years	4-years	5-years	6-years	7-years	8-years	9-years	10-years	11-years	12-years
AUC	0.70	0.75	0.74	0.76	0.77	0.79	0.79	0.77	0.77	0.76	0.75 6.08
Best threshold	6.14	6.34	5.93	6.36	6.37	6.13	6.37	6.06	6.03	6.03	
Sensitivity	64.77%	59.63%	77.01%	61.13%	61.71%	76.21%	63.92%	76.76%	77.15%	76.58%	71.06%
Specificity	67.05%	76.62%	56.58%	78.25%	78.79%	67.00%	79.02%	63.84%	62.76%	63.25%	65.98%

AUC, area under the time-dependent ROC curves.

## Discussion

In this longitudinal cohort study conducted on a large general population, we had the following important findings: (1) There was a significant and positive correlation between METS-VF, a novel indicator for assessing visceral adiposity, and diabetes risk, but this correlation may be non-linear, and when METS-VF exceeded the Q3 (5.58-6.00) interval, its correlation with diabetes risk was further enhanced. (2) METS-VF demonstrated significantly better performance compared to several other commonly used surrogate indicators of visceral adiposity, VAI, WC, WHtR, and BMI, in predicting future diabetes risk. (3) For the first time, we discovered that METS-VF exhibited higher AUC values and more stable predictive thresholds for predicting diabetes risk over the next 6-10 years, and was an ideal risk marker for future medium- to long-term diabetes risk.

The global prevalence of diabetes and obesity has shown an almost parallel increase in recent years, particularly in Asian populations, primarily in East Asia (6, 29). Importantly, epidemiological evidence indicates that the overall body fat content in Asian populations is typically lower compared to Western populations. However, abdominal obesity, characterized by the accumulation of fat around the abdomen, is a prominent feature of obesity in Asian populations (30–32). It is also a significant risk factor for metabolic disorders such as diabetes and cardiovascular disease (33, 34). In previous studies related to the mechanism of abdominal obesity leading to metabolic complications, most of them emphasized the importance of increased visceral fat rather than subcutaneous fat (35, 36), because subcutaneous adipose tissue is considered to be the largest and least metabolically harmful storage site for excess fat in the body (37), while deposition of excess adipose tissue such as ceramide or diacylglycerol in organs such as the liver and skeletal muscle will cause endocrine dysfunction, dysfunction of pro-inflammatory factors and mitochondrial dysfunction in visceral adipose tissue and also lead to increased levels of free fatty acids thereby antagonizing hepatic insulin (38–40). Given that abdominal subcutaneous adipose tissue and visceral adipose tissue may have opposite biological functions on the body’s glucose metabolism, accurate differentiation and measurement of visceral adipose tissue will help to assess and predict the occurrence and progression of diabetes.

Although MRI techniques and dual-energy X-ray absorptiometry (DXA) techniques can currently be used in clinical practice to accurately measure visceral fat content, the high economic and technical costs of these ancillary techniques make them unsuitable for use in primary health care and large-scale epidemiological investigations of diabetes (10). To address this issue, a large number of researchers are working to develop simple parameters that can more accurately identify and assess visceral adiposity. METS-VF is a new parameter for assessing visceral adiposity tissue developed and validated by Bello-Chavolla OY et al. in July 2019, and the detailed steps of its development and validation have been described elsewhere (12). Briefly, Bello-Chavolla OY et al. prospectively recruited a discovery cohort of 366 subjects with DXA measurements from healthcare institutions and used the visceral fat content of subjects obtained from DXA measurements as the dependent variable, and used several simple indicators (metabolic score for insulin resistance, age, sex, and WHtR), which are considered to be closely related to visceral fat content, as independent variables (41, 42), and then used non-linear regression analysis to fit the prediction model with the highest agreement with DXA measurements, namely METS-VF. METS-VF was subsequently validated by applying it to two validation cohorts of subjects with DXA+MRI measurements and subjects with bio-electrical impedance measurements, respectively. Their results showed that METS-VF was more accurate in predicting visceral adiposity than other obesity indicators such as WC, WHtR, VAI, and BMI both in the discovery cohort and in the validation cohort. In several subsequently published observational studies, cross-sectional data from Yu P et al. and longitudinal cohort data from Feng L et al. showed that METS-VF was significantly and independently positively associated with chronic kidney disease and had stronger risk assessment/predictive power for chronic kidney disease compared to other obesity indicators (13, 14). In addition, METS-VF has also been shown to be an independent predictor of hypertension and hyperuricemia (15, 16).

In the current study, we found a significant association between METS-VF and diabetes risk after adjusting for a large number of confounding factors associated with diabetes risk, with each unit increase in METS-VF increasing the risk of diabetes by 68%. Additionally, by observing Model 4 in **Table 2** and the dose-response curve in **Figure 3**, we found that there may be a non-linear correlation between METS-VF and diabetes risk, with a change in the correlation around the Q3 (5.58 to 6.00) interval of METS-VF, and a significantly stronger correlation with diabetes risk when METS-VF was located in the Q4 and Q5 intervals than in the Q1 and Q2 intervals. This finding was consistent with the findings of Feng Y et al. who also found a non-linear association between METS-VF and type 2 diabetes in a study of a rural population in Henan, China (17). Therefore, we recommend that both healthy and diabetic people should control their fat intake, body weight, and WC to keep METS-VF below the Q3 interval (METS-VF<6) as much as possible to minimize the risk of diabetes. Furthermore, in line with the conclusions of Bello-Chavolla OY et al, the results of the ROC analysis of the current study showed that METS-VF had a significantly better predictive performance for diabetes compared to WC, WHtR, VAI, and BMI (All *P<*0.05, DeLong test), which may be thanks to its higher predictive accuracy for visceral adiposity (12). It is worth mentioning that in the study by Feng Y et al., they found that although METS-VF had the highest AUC value (0.69) for predicting diabetes compared to other obesity indicators, the power of METS-VF was not significantly different from WC and WHtR for predicting diabetes (*P*=0.058) (17); this result may be related to the smaller study population and relatively short follow-up period (up to 6 years) of Feng Y et al.

Based on the longest 13-year follow-up data of 15,464 subjects, the current study used time-dependent ROC analysis to further explore the predictive power of METS-VF for the occurrence of diabetes at each time point over the next 2-12 years, showing that the predictive power of METS-VF for diabetes exhibited a slowly increasing trend from year 2 to year 7 of follow-up, while the highest predictive power was reached in years 7 and 8 (AUC=0.79), followed by a gradual decrease in the predictive power of METS-VF from year 9 to year 12. Therefore, it was more accurate to say that METS-VF should be more suitable for predicting future diabetes risk in the medium- and long-term (6-10 years), whereas the maximum 6-year follow-up period in the study by Feng Y et al. may have led them to underestimate the predictive power of METS-VF for future diabetes risk (17). Furthermore, it is worth noting that although the study by Feng Y et al. was also a longitudinal cohort study with follow-up, the time-dependence of diabetes status and METS-VF was not considered in their ROC analysis, which may also lead to some bias in their results (43). In summary, the results of the current study regarding the predictive power of METS-VF for the occurrence of diabetes in different future periods obtained by using time-dependent ROC analysis were more realistic and reliable (44). Given the higher predictive accuracy and more stable predictive thresholds of METS-VF for medium- and long-term diabetes risk, we recommended adding METS-VF to patients’ physical examination reports in primary health care and clinical practice as a novel risk marker for predicting future medium- and long-term diabetes risk and, meanwhile, we believed it was relatively safe to keep METS-VF below 6.

### Strengths and limitations

The strengths of the current study are the following: (1) the current study has a larger sample size (n=15,464) and a longer follow-up period (up to 13 years) of the general population cohort compared to the previous studies. (2) The current study explored the predictive power of baseline METS-VF for the occurrence of diabetes in different future periods using time-dependent ROC analysis, and for the first time, it was clear that METS-VF may be most suitable for predicting the risk of diabetes in the medium- and long-term (6-10 years), which provided a more accurate reference for the application of METS-VF for diabetes screening and prevention in primary health care.

This study also has the following limitations: (1) Subjects in the current study did not undergo MRI or DXA examinations to measure visceral fat mass, so we were unable to further compare the correlation between METS-VF and actual visceral fat mass and diabetes risk. (2) Diabetes was defined based on HbA1c ≥6.5% or FPG ≥7.0 mmol/L or subject self-report and did not include patients with abnormal 2-hour postprandial glucose, which may underestimate the correlation between METS-VF and diabetes risk. (3) The current study did not distinguish between types of diabetes, but considering that insulin resistance due to visceral adiposity is the pathogenesis of type 2 diabetes, and that type 2 diabetes accounts for more than 95% of all diabetes, and that type 1 diabetes and type 2 diabetes have different pathogenic characteristics, the results of the current study may be more applicable to type 2 diabetes (45, 46). (4) Although the current study adjusted a large number of confounding factors related to the risk of diabetes, there may still be some risk factors for diabetes that have not been adjusted due to it being an observational study, which may lead to some residual confounding. (5) The current study was a single-center cohort study, so the applicability of the findings to other ethnic populations will need to be further validated in future studies. (6) The current study did not repeat the measurement of all baseline indicators for the subjects during the follow-up period, which limited further exploration of the impact of dynamic changes in METS-VF on the risk of developing diabetes. This aspect needed to be further investigated in future studies.

## Conclusion

In conclusion, the current study demonstrated a significant positive correlation between METS-VF, a novel indicator for assessing visceral adiposity, and the risk of diabetes in the general population. Furthermore, compared to other surrogate indicators for general/visceral adiposity (BMI, WC, WHtR, VAI), baseline METS-VF had a better predictive performance for future diabetes risk and was particularly suitable for predicting future diabetes risk in the medium- and long-term.

## Data availability statement

The original contributions presented in the study are included in the article/**Supplementary Materials**. Further inquiries can be directed to the corresponding author.

## Ethics statement

The studies involving human participants were reviewed and approved by the Ethics Committee of Jiangxi Provincial People’s Hospital. Written informed consent for participation was not required for this study in accordance with the national legislation and the institutional requirements.

## Author contributions

YZ, RY, MK, JQ, CY and GS conceived the research, drafted the manuscript, and did the statistical analysis. YZ revised the manuscript and designed the study. All authors read and approved the final manuscript.
