# Supplementary material for: Assessing the usefulness of a newly proposed metabolic score for visceral fat in predicting future diabetes: results from the NAGALA cohort study
Source: Front Endocrinol (Lausanne). 2023 Jul 19;14:1172323. doi: 10.3389/fendo.2023.1172323 (PMC10395081; doi:10.3389/fendo.2023.1172323)
Supplement: Supplementary file 1 [file DataSheet_1.docx]

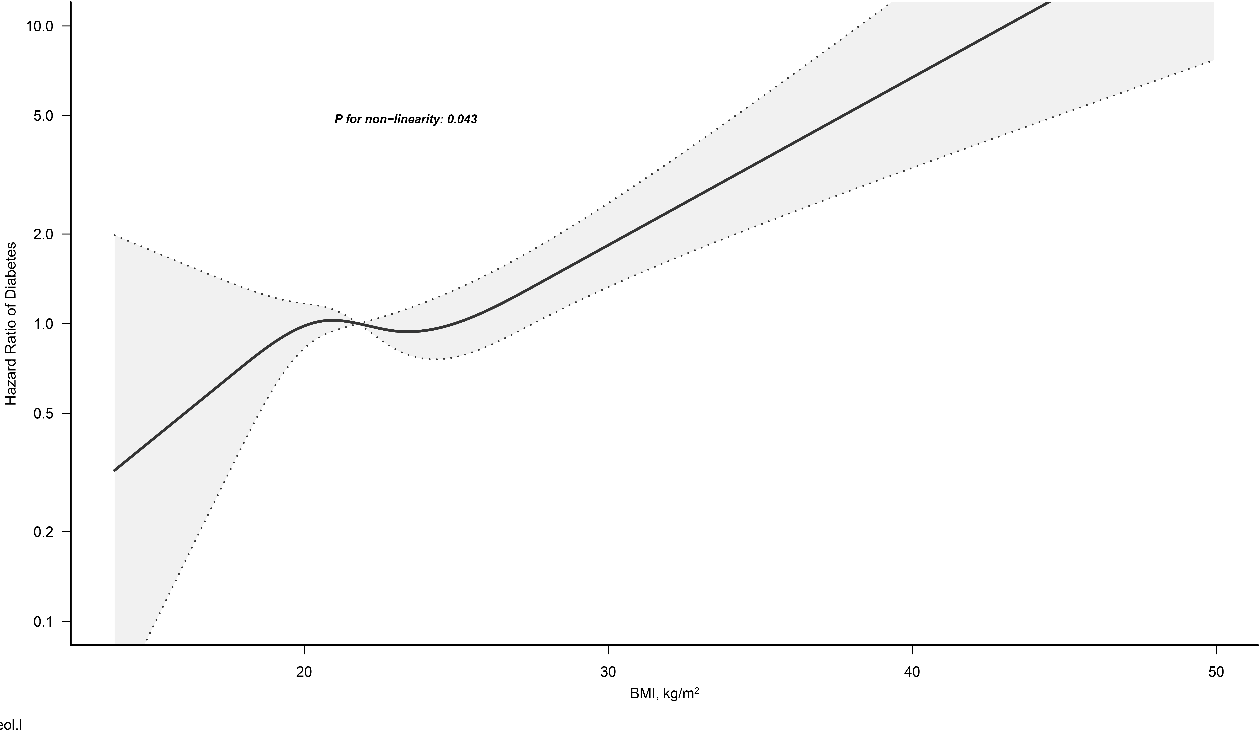
Supplementary figure 1: Restricted cubic spline analysis of BMI for the estimation of the risk of diabetes. Restricted cubic spline model adjusted for sex, age, fatty liver, height, exercise habits, ALT, AST, GGT, HDL-C, TC, TG, HbA1c, drinking status, smoking status, FPG, and SBP.


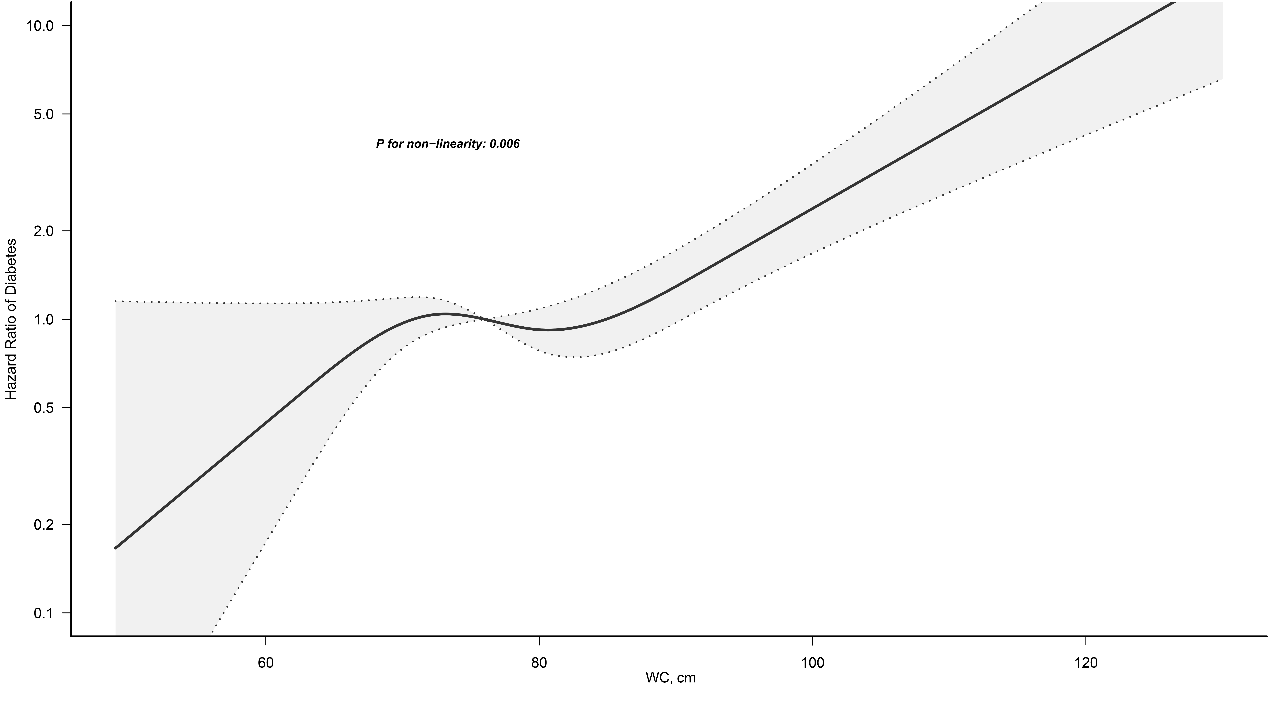
 Supplementary figure 2: Restricted cubic spline analysis of WC for the estimation of the risk of diabetes. Restricted cubic spline model adjusted for sex, age, fatty liver, height, exercise habits, ALT, AST, GGT, HDL-C, TC, TG, HbA1c, drinking status, smoking status, FPG, and SBP.


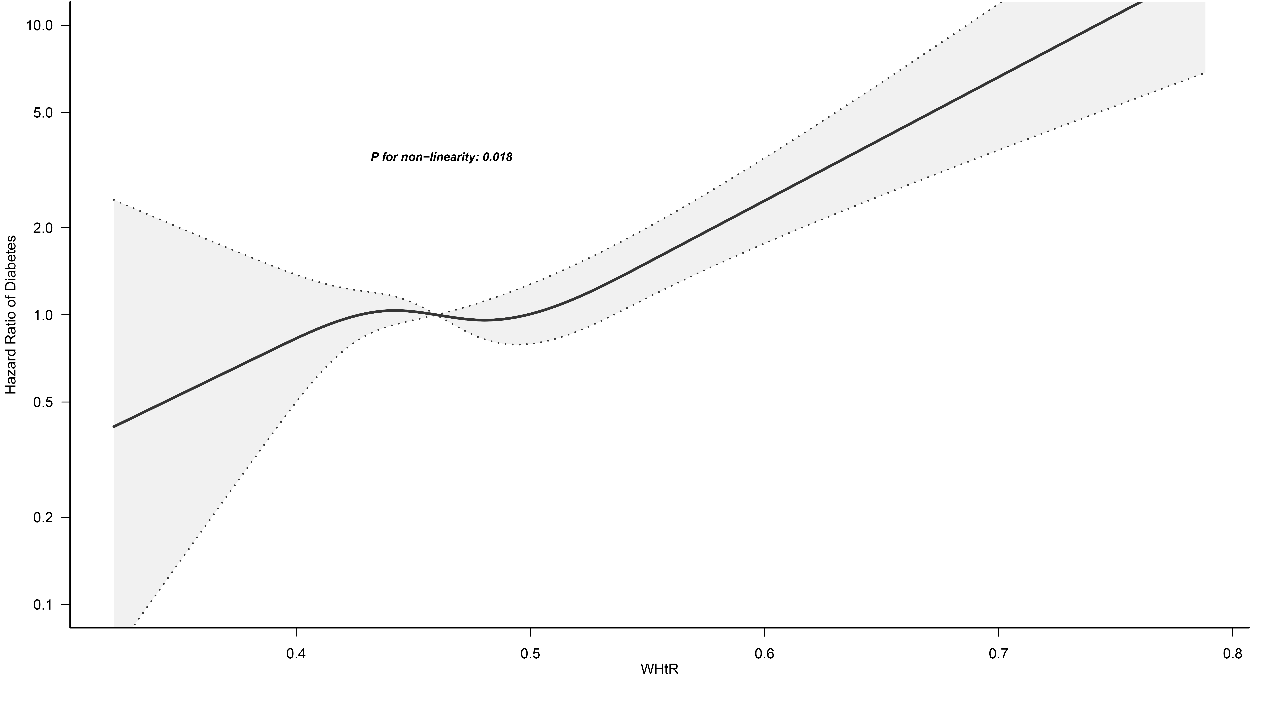
 Supplementary figure 3: Restricted cubic spline analysis of WHtR for the estimation of the risk of diabetes. Restricted cubic spline model adjusted for sex, age, fatty liver, height, exercise habits, ALT, AST, GGT, HDL-C, TC, TG, HbA1c, drinking status, smoking status, FPG, and SBP.


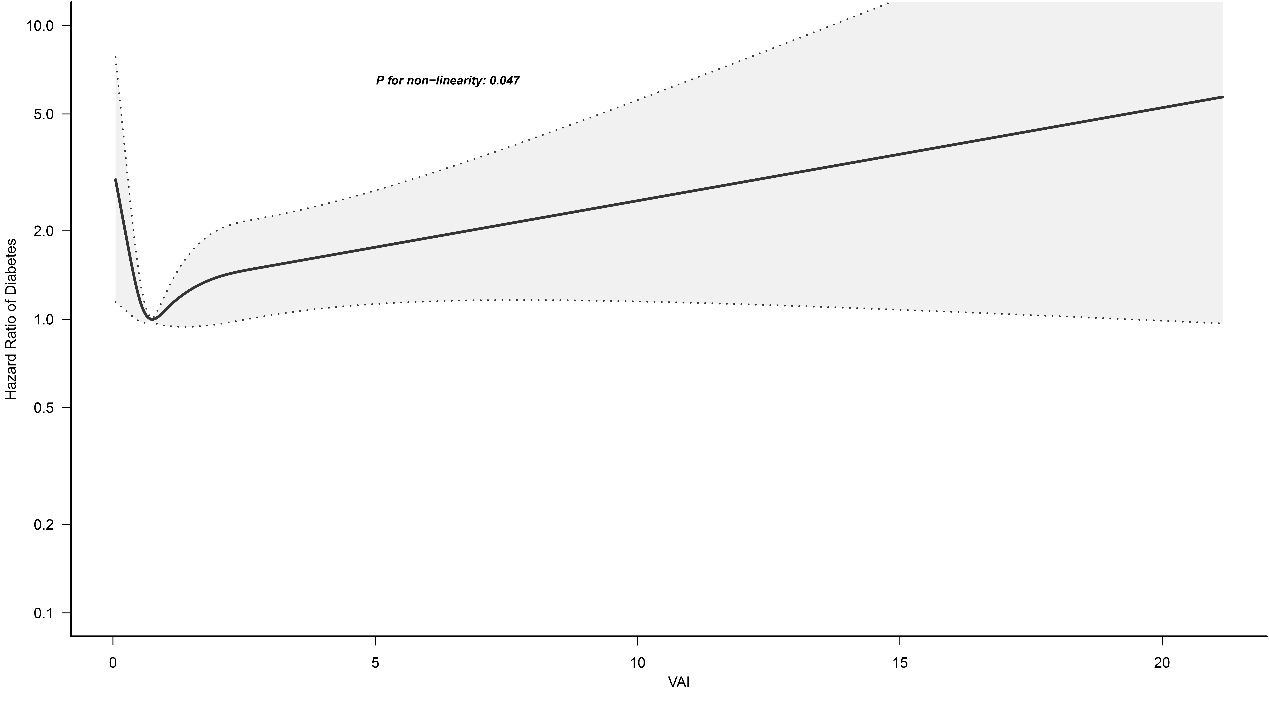
 Supplementary figure 4: Restricted cubic spline analysis of VAI for the estimation of the risk of diabetes. Restricted cubic spline model adjusted for sex, age, fatty liver, height, BMI, exercise habits, ALT, AST, GGT, HDL-C, TC, HbA1c, drinking status, smoking status, FPG, and SBP.
