# Supplementary material for: Assessing the usefulness of a newly proposed metabolic score for visceral fat in predicting future diabetes: results from the NAGALA cohort study
Source: Front Endocrinol (Lausanne). 2023 Jul 19;14:1172323. doi: 10.3389/fendo.2023.1172323 (PMC10395081; doi:10.3389/fendo.2023.1172323)
Supplement: Supplementary file 4 [file Table_3.docx]

Supplementary Table 1: Univariate Cox regression analysis of the associations between diabetes and baseline variables.

|  | HR (95%CI) *P*-value |
| --- | --- |
| Sex |  |
| women | 1.0 |
| men | 2.52 (1.98, 3.21) <0.0001 |
| Age | 1.06 (1.04, 1.07) <0.0001 |
| Height | 6.90 (2.02, 23.50) 0.0020 |
| Weight | 1.06 (1.05, 1.06) <0.0001 |
| BMI | 1.24 (1.22, 1.27) <0.0001 |
| WC | 1.09 (1.08, 1.10) <0.0001 |
| Exercise habits |  |
| None | 1.0 |
| Yes | 0.76 (0.56, 1.02) 0.0641 |
| ALT | 1.01 (1.01, 1.01) <0.0001 |
| AST | 1.01 (1.01, 1.01) <0.0001 |
| GGT | 1.01 (1.01, 1.01) <0.0001 |
| METS-VF | 5.14 (4.27, 6.19) <0.0001 |
| HDL-C | 0.13 (0.09, 0.18) <0.0001 |
| TC | 1.49 (1.34, 1.66) <0.0001 |
| TG | 1.80 (1.68, 1.92) <0.0001 |
| FPG | 1.20 (1.18, 1.22) <0.0001 |
| HbA1c | 54.27 (39.49, 74.59) <0.0001 |
| Fatty liver | 7.02 (5.70, 8.63) <0.0001 |
| Drinking status |  |
| Non/small | 1.0 |
| Light | 0.90 (0.65, 1.26) 0.5508 |
| Moderate | 1.15 (0.82, 1.62) 0.4240 |
| Heavy | 2.24 (1.54, 3.27) <0.0001 |
| Smoking status |  |
| None | 1.0 |
| Past | 1.65 (1.26, 2.18) 0.0004 |
| Current | 2.58 (2.06, 3.24) <0.0001 |
| SBP | 1.03 (1.03, 1.04) <0.0001 |
| DBP | 1.05 (1.04, 1.06) <0.0001 |

Abbreviations: HR: Hazard ratio; CI: confidence interval; other abbreviations as in Table 1.

Supplementary Table 2: Collinearity screening between METS-VF and other variables.

|  | Variance inflation factor | | | |
| --- | --- | --- | --- | --- |
|  | Step 1 | Step 2 | Step 3 | Step 4 |
| METS-VF | 21.1 | 21.1 | 5.2 | 5.2 |
| Sex | 4.4 | 4.4 | 3.5 | 3.4 |
| Age | 1.9 | 1.9 | 1.7 | 1.6 |
| Fatty liver | 1.6 | 1.6 | 1.6 | 1.6 |
| Height | 54.4 | 6.1 | 2.4 | 2.4 |
| Weight | 170.9 | NA | NA | NA |
| BMI | 96.9 | 5.1 | 4.2 | 4.2 |
| WC | 24.4 | 24.3 | NA | NA |
| Exercise | 1 | 1 | 1 | 1 |
| ALT | 4.2 | 4.1 | 4.1 | 4.1 |
| AST | 3.3 | 3.3 | 3.3 | 3.3 |
| GGT | 1.5 | 1.5 | 1.5 | 1.5 |
| FPG | 1.5 | 1.5 | 1.5 | 1.5 |
| HDL-C | 1.9 | 1.9 | 1.9 | 1.9 |
| TC | 1.4 | 1.4 | 1.4 | 1.4 |
| TG | 1.8 | 1.8 | 1.8 | 1.8 |
| HbA1c | 1.3 | 1.3 | 1.3 | 1.3 |
| Drinking status | 1.3 | 1.3 | 1.3 | 1.3 |
| Smoking status | 1.4 | 1.4 | 1.4 | 1.4 |
| SBP | 5.6 | 5.6 | 5.6 | 1.4 |
| DBP | 5.7 | 5.7 | 5.7 | NA |

Note-1: Variance inflation factor = 1/(1-R^2^). Abbreviations as in Table 1.

Note-2: The variables with variance inflation factor >5 will be regarded as collinear variables and cannot be included in the multiple regression model.

Supplementary Table 3: Collinearity screening between BMI and other variables.

|  | Variance inflation factor | | | | |
| --- | --- | --- | --- | --- | --- |
|  | Step 1 | Step 2 | Step 3 | Step 4 | Step 5 |
| BMI | 97 | 5.1 | 4.4 | 4.4 | 1.8 |
| Sex | 4.6 | 4.6 | 3.5 | 3.4 | 3.2 |
| Age | 2 | 2 | 1.7 | 1.7 | 1.3 |
| Fatty liver | 1.6 | 1.6 | 1.6 | 1.6 | 1.6 |
| Height | 54.5 | 6.4 | 2.4 | 2.4 | 2.4 |
| Weight | 171 | NA | NA | NA | NA |
| WC | 26 | 25.9 | NA | NA | NA |
| Exercise | 1 | 1 | 1 | 1 | 1 |
| ALT | 4.2 | 4.1 | 4.1 | 4.1 | 4.1 |
| AST | 3.3 | 3.3 | 3.3 | 3.3 | 3.3 |
| GGT | 1.5 | 1.5 | 1.5 | 1.5 | 1.5 |
| METS-VF | 23.4 | 23.3 | 5.4 | 5.4 | NA |
| HDL-C | 1.9 | 1.9 | 1.9 | 1.9 | 1.8 |
| TC | 1.4 | 1.4 | 1.4 | 1.4 | 1.4 |
| TG | 1.8 | 1.8 | 1.8 | 1.8 | 1.8 |
| HbA1c | 1.3 | 1.3 | 1.3 | 1.3 | 1.2 |
| Drinking status | 1.3 | 1.3 | 1.3 | 1.3 | 1.3 |
| Smoking status | 1.4 | 1.4 | 1.4 | 1.4 | 1.4 |
| FPG | 1.5 | 1.5 | 1.5 | 1.5 | 1.5 |
| SBP | 5.6 | 5.6 | 5.6 | 1.4 | 1.4 |
| DBP | 5.7 | 5.7 | 5.7 | NA | NA |

Note-1: Variance inflation factor = 1/(1-R^2^). Abbreviations as in Table 1.

Note-2: The variables with variance inflation factor >5 will be regarded as collinear variables and cannot be included in the multiple regression model.

Supplementary Table 4: Collinearity screening between WC and other variables.

|  | Variance inflation factor | | | | |
| --- | --- | --- | --- | --- | --- |
|  | Step 1 | Step 2 | Step 3 | Step 4 | Step 5 |
| WC | 26 | 25.9 | 6 | 6 | 2.1 |
| Sex | 4.6 | 4.6 | 3.2 | 3.2 | 3.2 |
| Age | 2 | 2 | 1.4 | 1.4 | 1.3 |
| Fatty liver | 1.6 | 1.6 | 1.6 | 1.6 | 1.5 |
| Height | 54.5 | 6.4 | 2.8 | 2.8 | 2.5 |
| Weight | 171 | NA | NA | NA | NA |
| BMI | 97 | 5.1 | 5.1 | 5.1 | NA |
| Exercise | 1 | 1 | 1 | 1 | 1 |
| ALT | 4.2 | 4.1 | 4.1 | 4.1 | 4.1 |
| AST | 3.3 | 3.3 | 3.3 | 3.3 | 3.3 |
| GGT | 1.5 | 1.5 | 1.5 | 1.5 | 1.5 |
| METS-VF | 23.4 | 23.3 | NA | NA | NA |
| HDL-C | 1.9 | 1.9 | 1.8 | 1.8 | 1.8 |
| TC | 1.4 | 1.4 | 1.4 | 1.4 | 1.4 |
| TG | 1.8 | 1.8 | 1.8 | 1.8 | 1.8 |
| HbA1c | 1.3 | 1.3 | 1.3 | 1.3 | 1.3 |
| Drinking status | 1.3 | 1.3 | 1.3 | 1.3 | 1.3 |
| Smoking status | 1.4 | 1.4 | 1.4 | 1.4 | 1.4 |
| FPG | 1.5 | 1.5 | 1.5 | 1.5 | 1.5 |
| SBP | 5.6 | 5.6 | 5.6 | 1.4 | 1.4 |
| DBP | 5.7 | 5.7 | 5.7 | NA | NA |

Note-1: Variance inflation factor = 1/(1-R^2^). Abbreviations as in Table 1.

Note-2: The variables with variance inflation factor >5 will be regarded as collinear variables and cannot be included in the multiple regression model.

Supplementary Table 5: Collinearity screening between WHtR and other variables.

|  | Variance inflation factor | | | | |
| --- | --- | --- | --- | --- | --- |
|  | Step 1 | Step 2 | Step 3 | Step 4 | Step 5 |
| WHtR | 20.5 | 20.3 | 4.7 | 4.7 | 1.7 |
| Sex | 4.6 | 4.5 | 3.2 | 3.2 | 3.2 |
| Age | 2 | 2 | 1.4 | 1.4 | 1.3 |
| Fatty liver | 1.6 | 1.6 | 1.6 | 1.6 | 1.5 |
| Height | 52.6 | 2.5 | 2.5 | 2.5 | 2.4 |
| Weight | 172.3 | NA | NA | NA | NA |
| BMI | 100.4 | 5 | 5 | 5 | NA |
| Exercise | 1 | 1 | 1 | 1 | 1 |
| ALT | 4.2 | 4.1 | 4.1 | 4.1 | 4.1 |
| AST | 3.3 | 3.3 | 3.3 | 3.3 | 3.3 |
| GGT | 1.5 | 1.5 | 1.5 | 1.5 | 1.5 |
| METS-VF | 23.3 | 23.2 | NA | NA | NA |
| HDL-C | 1.9 | 1.9 | 1.8 | 1.8 | 1.8 |
| TC | 1.4 | 1.4 | 1.4 | 1.4 | 1.4 |
| TG | 1.8 | 1.8 | 1.8 | 1.8 | 1.8 |
| HbA1c | 1.3 | 1.3 | 1.3 | 1.3 | 1.3 |
| Drinking status | 1.3 | 1.3 | 1.3 | 1.3 | 1.3 |
| Smoking status | 1.4 | 1.4 | 1.4 | 1.4 | 1.4 |
| FPG | 1.5 | 1.5 | 1.5 | 1.5 | 1.5 |
| SBP | 5.6 | 5.6 | 5.6 | 1.4 | 1.4 |
| DBP | 5.7 | 5.7 | 5.7 | NA | NA |

Note-1: Variance inflation factor = 1/(1-R^2^). Abbreviations as in Table 1.

Note-2: The variables with variance inflation factor >5 will be regarded as collinear variables and cannot be included in the multiple regression model.

Supplementary Table 6: Collinearity screening between VAI and other variables.

|  | Variance inflation factor | | | | |
| --- | --- | --- | --- | --- | --- |
|  | Step 1 | Step 2 | Step 3 | Step 4 | Step 5 |
| VAI | 12.4 | 12.4 | 1.8 | 1.8 | 1.8 |
| Sex | 4.1 | 4.1 | 3.6 | 3.6 | 3.3 |
| Age | 1.7 | 1.7 | 1.7 | 1.7 | 1.3 |
| Fatty liver | 1.6 | 1.6 | 1.6 | 1.6 | 1.6 |
| Height | 52.4 | 2.5 | 2.4 | 2.4 | 2.4 |
| Weight | 170.3 | NA | NA | NA | NA |
| BMI | 97 | 4.4 | 4.4 | 4.4 | 1.8 |
| Exercise | 1 | 1 | 1 | 1 | 1 |
| ALT | 4.2 | 4.1 | 4.1 | 4.1 | 4.1 |
| AST | 3.3 | 3.3 | 3.3 | 3.3 | 3.3 |
| GGT | 1.5 | 1.5 | 1.5 | 1.5 | 1.5 |
| METS-VF | 5.5 | 5.5 | 5.5 | 5.5 | NA |
| HDL-C | 2.3 | 2.3 | 2.1 | 2.1 | 2.1 |
| TC | 1.5 | 1.5 | 1.4 | 1.4 | 1.4 |
| TG | 12.3 | 12.3 | NA | NA | NA |
| HbA1c | 1.3 | 1.3 | 1.3 | 1.3 | 1.2 |
| Drinking status | 1.3 | 1.3 | 1.3 | 1.3 | 1.3 |
| Smoking status | 1.4 | 1.4 | 1.4 | 1.4 | 1.4 |
| FPG | 1.5 | 1.5 | 1.5 | 1.5 | 1.5 |
| SBP | 5.6 | 5.6 | 5.6 | 1.4 | 1.4 |
| DBP | 5.7 | 5.7 | 5.7 | NA | NA |

Note-1: Variance inflation factor = 1/(1-R^2^). Abbreviations as in Table 1.

Note-2: The variables with variance inflation factor >5 will be regarded as collinear variables and cannot be included in the multiple regression model.
